# Pregnancy in Noncommunicating Rudimentary Horn of Unicornuate Uterus: A Case Report and Review of the Literature

**DOI:** 10.1155/2019/1489751

**Published:** 2019-12-14

**Authors:** Melese Gezahegn Tesemma

**Affiliations:** Arsi University, College of Health Science, Department of Obstetrics & Gynecology, Asella, Ethiopia

## Abstract

Pregnancy implanted in the rudimentary horn of unicornuate uterus is very rare with an incidence of 1 in 75,000 to 1 in 150,000 pregnancies. Both prerupture and postrupture diagnosis of ectopic pregnancy in the rudimentary horn of a unicornuate uterus is difficult. Here is a case of a 21-year-old gravida 5 para 3 abortion 1 mother presented with abdominal pain of 2 days duration following a falling accident. The patient was severely anemic and was in hypovolemic shock at the time of presentation. She had diffused lower abdominal tenderness with hemoperitonium. After clinical and ultrasound evaluation, emergency laparotomy was decided for preop diagnosis of ruptured cornual ectopic pregnancy to rule out uterine rupture at gestational age of 16 weeks. Intraoperatively, ruptured ectopic pregnancy in noncommunicating rudimentary horn was diagnosed. Resection of rudimentary horn and ipsilateral salpingectomy was done. She was transfused with 5 units of compatible blood. It is better to increase awareness about pregnancy occurring in this rare uterine anomaly, so as to have a high index of suspicion as early detection before it gets ruptured is difficult.

## 1. Introduction

A unicornuate uterus with a rudimentary horn results from incomplete development of one of the Müllerian ducts and an incomplete fusion with the contralateral side [1–3]. The incidence of pregnancy occurring in a rudimentary horn of the unicornuate uterus is very rare ranging from 1 in 75,000 to 150,000 pregnancies. Usually the diagnosis is difficult and sometimes missed as the majority of the cases present on emergency with hemoperitonium [2, 4, 5]. About 85% of rudimentary uterine horns are noncommunicating in type [1, 5]. Ectopic pregnancy in this extremely rare anomaly ends up with rupture during all trimesters of pregnancy. Diagnosis of ectopic pregnancy in rudimentary horn is difficult, especially in a woman with a prior vaginal delivery. Thus a high index of suspicion is needed as in the majority of cases it is detected after it gets ruptured [3, 6]. Ectopic pregnancy in the rudimentary horn of unicornuate uterus is associated with life threatening complications, one of which is uterine rupture with a 50% risk [4, 5]. Here, I report a case of ectopic pregnancy in the rudimentary horn of a unicornuate uterus at gestational age of 16 weeks.

## 2. Case Report

A 21-year-old gravida 5 para 3 (all alive) abortion 1 mother with a pregnancy of 4 months was presented with severe lower abdominal pain of 2 days duration following a falling accident on her abdomen 5 days ago. She had easy fatigability, vertigo, and light headedness since two days after the accident, but had no vaginal bleeding, fever, headache, chills, and vaginal discharge and she did not feel fetal movement yet. She had no antenatal care follow up. She was referred from a nearby primary hospital to the Jimma university medical center with diagnosis of septic shock secondary to septic abortion where she was admitted and given antibiotics. Medical abortion was tried, but failed at the primary hospital. She had no personal or familial history of hypertension and diabetes. She had no history of chronic pelvic pain. She had no history of ectopic pregnancy, but had one history of spontaneous abortion which occurred at the 4^th^ month. Prior to this accident, she had no history of abdominal pain, bloating, and epigastric pain.

On physical examination, she was acutely sick looking, agitated, and confused in appearance. Her vital signs were as follows: Blood Pressure: 70/30 mmHg, PR: 148 bpm, RR: 24 br/m, T^0^: 37.5. Her conjunctiva was paper white and had dry tongue and buccal mucosa. Chest was clear and resonant. S3 gallop was heard. On the abdomen there was diffuse lower abdominal tenderness and guarding with positive signs of fluid collection. On pelvic examination cervix was closed with no blood on the examining finger, but, had a right adnexal tenderness.

On ultrasound exam, empty uterus was seen. A collapsed fetus with no cardiac activity having femur length measurement corresponding to 16 weeks and placenta measuring 4 cm × 5 cm looks attached to the left cornual part of the uterus was seen. There was significant free fluid in cul-de-sac and paracolic gutter. Otherwise liver and both kidneys were grossly normal. With impression of hypovolemic shock secondary to acute blood loss secondary to ruptured cornual ectopic pregnancy to rule out uterine rupture and severe anemia. The patient was resuscitated with 3 liters of intravenous crystalloids while preparing for an emergency laparotomy. She was investigated for blood group and Rh (A+) and complete blood count. Hematocrit was 6%, RBC count was only 0.7 million, platelet count was 52,000, WBC count was 11,000, and neutrophils was 85%. Four units of cross-matched blood were prepared before taking to the operation theatre.

**Figure 1:**
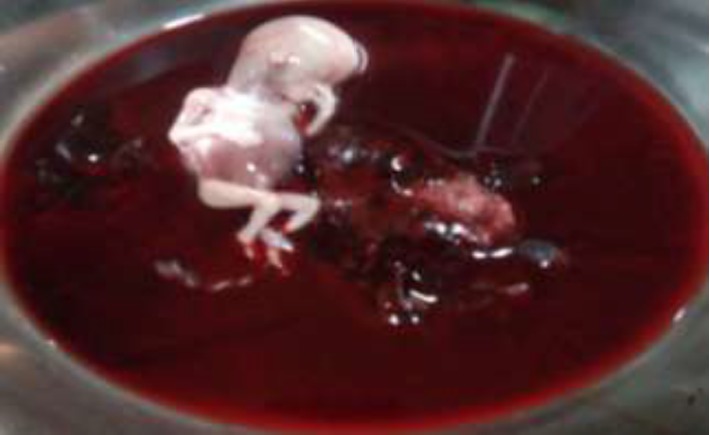
Dead male fetus and placenta removed from the peritoneal cavity.

Intraoperatively there was about 2500 ml hemoperitonium, ruptured left side rudimentary horn ectopic pregnancy, dead male fetus weighing about 300 gm (Figure 1), and the placenta partly attached to the cavity of the rudimentary horn. The unicornuate uterus with a normal ovary and tube was seen. The rudimentary horn had a cavity which was noncommunicating with the unicornuate uterine cavity and it was attached to the unicornuate uterus by fibrous tissue. The left round ligament and the ovary are directly attached to the rudimentary horn rather than the unicornuate uterus.

**Figure 2:**
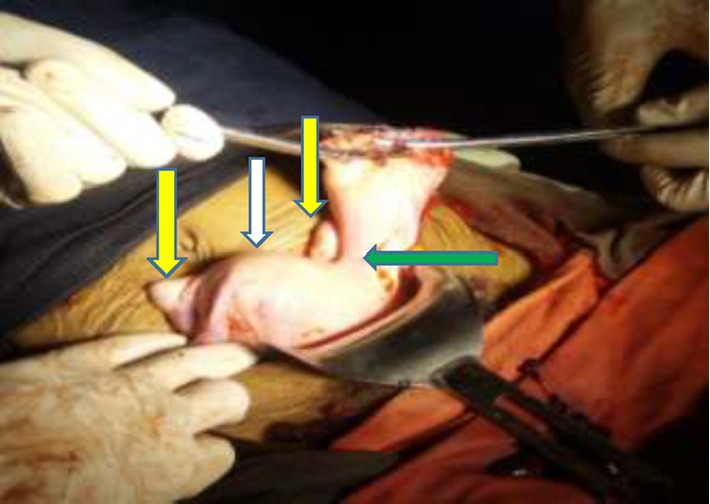
Uni-cornuate uterus (white arrow) with left side rudimentary horn (green arrow) attached to uterine body by dense fibro-muscular tissue. Ovaries are bilaterally visible (yellow arrows).

**Figure 3:**
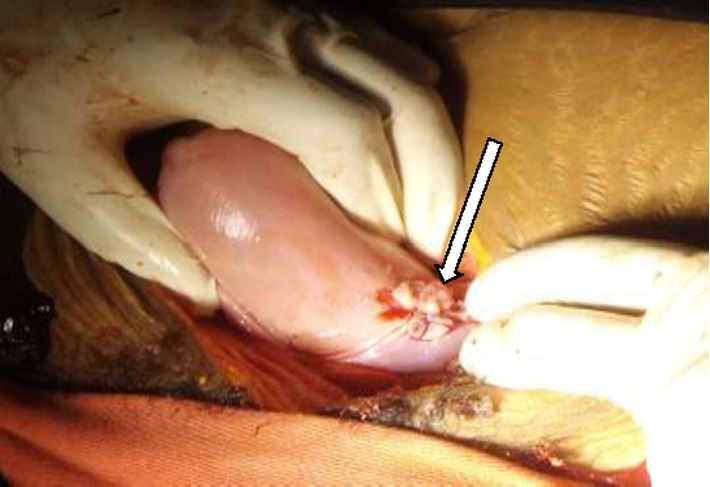
Stump (arrow) of the resected rudimentary horn after resection done.

Hemoperitonium was sucked out, the fetus and the placenta was removed. The round ligament and the utero ovarian ligament of the horn was clamped, cut, and ligated. The ruptured rudimentary horn was clamped and resected at its base and left side salpingectomy was done (Figure 2). The base was ligated to secure hemostasis and finally the stump was checked for any bleeding. (Figure 3). At the end, the abdominal cavity was mopped and closed in layers. The patient was transfused with 2 units of compatible blood intraoperatively and the other two on the immediate post-op day. Intraoperatively, a diagnosis of ruptured ectopic pregnancy in noncommunicating rudimentary horn of unicornuate uterus was made.

After being transfused with a total of 5 units of blood post-op, hematocrit increased to 30%, and symptoms of anemia improved. Post-operative condition was smooth and was finally discharged on the 6^th^ post-op day after providing contraception and with an advice to continue her ferrous sulfate and to come early for an ultrasound evaluation in subsequent pregnancies to prevent the recurrence risk of ectopic pregnancy.

## 3. Discussion

Mechanism of pregnancy occurrence in the noncommunicating rudimentary horn is assumed to be by transperitoneal migration of either the fertilized ovum or the spermatozoon from the contralateral tube [2, 7, 8]. Despite the rarity and its diagnostic challenge, such type of ectopic pregnancy is associated with severe fetal-maternal morbidities [7, 8]. This was true in our case where the diagnosis of rudimentary horn ectopic pregnancy in unicornuate was missed before laparotomy and the patient presented with hypovolemic shock and severe anemia necessitating massive transfusion. Thus creating awareness on this clinical presentation is very important so as to increase the rate of early diagnosis before rupture and prevent catastrophic adverse maternal outcomes [8, 9]. The natural fate of ectopic pregnancy in rudimentary horn is usually rupture during the last two trimesters due to underdevelopment, variable thickness, and poor distensibility of myometrium and dysfunctional endometrium. As a result very few (10%) of these pregnancies reach full term out of which only 2% of the fetuses can survive [10].

Although antenatal diagnosis of pregnancy in rudimentary horn is still tricky, advanced first trimester scanning may provide a clue for early diagnosis [4, 9]. But only few cases of rudimentary horn pregnancy were diagnosed in early trimester prior to rupture so far [8, 11]. Whenever ultrasound finding remains inconclusive one may consider magnetic resonance imaging due to its high soft tissue definition and confirmatory information [8–10]. Even though the sensitivity of ultrasonography to diagnose pregnancy in rudimentary horn is only 26–30% [3, 6], it would be helpful to use the three sonographic criterias suggested by Tsafrir and his associates for the diagnosis of rudimentary horn pregnancy [8].

Placement of a Foley catheter into the uterine cavity with filled balloon and performing a transabdominal ultrasound scan can conclusively exclude an intrauterine pregnancy as it is shown in one case report [11]. Foley catheter was also used to see whether the horn is communicating with the uterine cavity [10]. In our case, although the presence of extra uterine pregnancy was confirmed before laparotomy the fact that she sustained a falling accident and the presence of a fetus outside the uterine cavity increased our doubt of uterine rupture.

Resection of the rudimentary horn and the ipsilateral fallopian tube by either laparotomy or laparoscopy is the mainstay of the management of rudimentary horn ectopic pregnancy [1, 4, 8]. In our case, we resected the rudimentary horn with its fallopian tube by laparotomy. It is recommended not to postpone surgery once the diagnosis of a unruptured ectopic pregnancy in rudimentary horn is made as the timing of rupture depends on the thickness of the horn musculature and once it ruptures it leads to catastrophic complications [9]. Patients with unicornuate uterus with a rudimentary horn should be investigated for urinary anomalies; as imaging in some patients revealed an absent kidney on the ipsilateral side [7]. In our case, both kidneys were grossly normal on ultrasound. On discharge the patients should be advised on future risk of ectopic pregnancy and preterm birth related to this anomaly as it was addressed in our case [7].

## 4. Conclusion

There is a need for increased awareness of this rare anomaly and having a high index of suspicion, especially in developing countries where the possibility of early detection before rupture is difficult.
